# Linking Chemical Profile to Enzyme Inhibition: A Comprehensive Bio-Guided Study of *Lippia origanoides* Kunth Essential Oil

**DOI:** 10.3390/plants15081158

**Published:** 2026-04-09

**Authors:** Marta Pavarino, Cecilia Cagliero, Arianna Marengo, Carlo Bicchi, Francisco C. M. Chaves, Patrizia Rubiolo, Humberto R. Bizzo, Barbara Sgorbini

**Affiliations:** 1 Department of Drug Science and Technology, University of Turin, Via Pietro Giuria 9, 10125 Turin, Italy; m.pavarino@unito.it (M.P.); cecilia.cagliero@unito.it (C.C.); arianna.marengo@unito.it (A.M.); carlo.bicchi@unito.it (C.B.); patrizia.rubiolo@unito.it (P.R.); 2 Embrapa Food Technology, Avenida das Américas 29501, Rio de Janeiro 23020-470, RJ, Brazil; 3 Embrapa Western Amazon, Rodovia AM 010, km 29, Manaus 69010-970, AM, Brazil; celio.chaves@embrapa.br

**Keywords:** *Lippia origanoides* Kunth essential oil, Verbenaceae, chiral analysis, acetylcholinesterase, butyrylcholinesterase, α-glucosidase, lipase, tyrosinase, antioxidant, bio-guided fractionation

## Abstract

*Lippia origanoides* Kunth (Verbenaceae family), popularly known in northern Brazil as “Salva-de-Marajó”, is a native plant widely used in traditional medicine and cooking. While previous studies have addressed its antimicrobial and insecticidal properties, its ability to inhibit disease-related enzymes has received limited attention. This study investigated the essential oil (EO) of *L. origanoides* as a source of enzyme inhibitors relevant to Alzheimer’s disease, metabolic disorders and skin pigmentation disorders. The EO showed strong inhibitory activity against acetylcholinesterase (IC_50_: 22.9 μg/mL) and α-glucosidase (IC_50_: 14.6 μg/mL), indicating potential for managing neurodegenerative conditions and diabetes, respectively. Moderate inhibition was observed for lipase, butyrylcholinesterase and tyrosinase. Although carvacrol, the major EO constituent, contributed significantly to these effects, it did not fully explain the observed bioactivity. Bio-guided fractionation revealed that oxygenated compounds were mainly responsible for inhibiting cholinesterases and lipase, whereas α-glucosidase inhibition was associated with hydrocarbon compounds. Both fractions contributed to tyrosinase inhibition, reinforcing the EO’s relevance for treating hyperpigmentation. Furthermore, the EO demonstrated strong antioxidant activity, largely linked to carvacrol and oxygenated constituents. Chemical characterization by GC-MS, GC-FID and enantiomeric analysis strengthened the relationship between composition and bioactivity. Overall, *L. origanoides* EO emerged as a promising multifunctional natural product for therapeutic and cosmetic applications.

## 1. Introduction

*Lippia origanoides* Kunth is an aromatic shrub belonging to the Verbenaceae family widely used in Brazilian cuisine and traditional medicine, and it has gained increasing attention due to its diverse biological activities. Across Brazil, the plant, popularly known as “Salva-de-Marajó” (Marajó sage), is traditionally employed to treat stomach pain, indigestion, nausea, menstrual cramps, headaches, anxiety and various infectious conditions [1,2]. It also serves as a general antiseptic for affections of the mouth, throat and vagina; for wound care; and more broadly in the management of infectious diseases [3,4]. In Oriximiná (Pará State, Brazil), Quilombola women prepare decoctions and use vapors from the aerial parts orally or in sitz baths to alleviate dysmenorrhea symptoms [2]. *L. origanoides* essential oil (LOEO) is additionally valued in traditional medicine for its antimicrobial and anti-inflammatory effects [5].

Growing interest in this species reflects the broader search for natural, safe and multifunctional bioactive compounds for pharmaceutical, food and cosmetic applications. Essential oils (EOs) are complex mixtures of volatile plant metabolites long used in perfumery, cosmetics and pharmaceuticals [6,7]. Their potential to inhibit disease-related enzymes has become particularly relevant given the ongoing demand for innovative, multi-target natural substances.

This need is exemplified by conditions in which enzymatic dysregulation plays a key role. Diabetes mellitus and Alzheimer’s disease (AD), both highly prevalent among the elderly, share pathological mechanisms such as mitochondrial dysfunction [8]. Insulin resistance, a hallmark of diabetes, can influence AD pathology by altering amyloid precursor protein (APP) metabolism and promoting amyloid plaque formation [9]. Disturbances in glucose and lipid metabolism, often linked to obesity, further exacerbate AD progression, while gut microbiota alterations have also been implicated in obesity-related diseases [10].

Consequently, enzymes such as acetylcholinesterase (AChE), butyrylcholinesterase (BChE), α-glucosidase and lipase are considered promising therapeutic targets.

Enzymatic modulation is also of growing interest in dermatology, particularly in the context of melanin biosynthesis. Tyrosinase inhibition represents a central strategy for controlling excessive melanin production in hyperpigmentation disorders. Although tyrosinase inhibitors are widely used in clinical and cosmetic formulations, safety concerns have led to restrictions on compounds such as hydroquinone and monobenzyl ether hydroquinone under EU cosmetic regulation 1223/2009, while others, including citral, remain under evaluation by the SCCS due to potential side effects [11]. These limitations underscore the ongoing demand for new plant-derived molecules with safer profiles.

Oxidative stress is another shared factor linking neurodegeneration, metabolic dysfunction and skin aging. Antioxidants are therefore central to mitigating the progression of AD and diabetes, as well as preventing hyperpigmentation and skin damage. Given their chemical diversity and bioactivity, essential oils, particularly from species with significant ethnopharmacological use, are promising sources of multifunctional agents.

Previous studies on *L. origanoides* EO have confirmed some of its traditional uses. It has been shown to exert tocolytic effects and inhibit writhing in a dysmenorrhea mouse model [4], as well as exhibiting antimicrobial, insecticidal, acaricidal and larvicidal activities [12,13,14,15]. New pharmacological activities have also been reported, including anticonvulsant [16], spasmolytic [4], anesthetic [17], anxiolytic, and cardiovascular-related activities [18] and antivenom effects [19]. Despite this broad pharmacological potential, only two studies have examined its enzyme-inhibitory properties, both focusing solely on AChE inhibition [20,21].

In this study, a comprehensive chemical characterization of *L. origanoides* essential oil was carried out, including chiral analysis, and its inhibitory effects on AChE, BChE, α-glucosidase, lipase and tyrosinase, alongside its antioxidant capacity, were evaluated. Particular attention was given to the major constituent carvacrol to determine whether it alone accounts for the observed biological activity. This involved comparing expected inhibitory and antioxidant values, assuming carvacrol as the sole active compound, with experimentally observed outcomes, and applying, for the first time, a bio-guided fractionation approach to identify the compounds responsible for the EOs’ multifaceted bioactivity.

## 2. Results

### 2.1. Chemical Composition of Lippia origanoides Kunth Essential Oil

**Figure 1:**
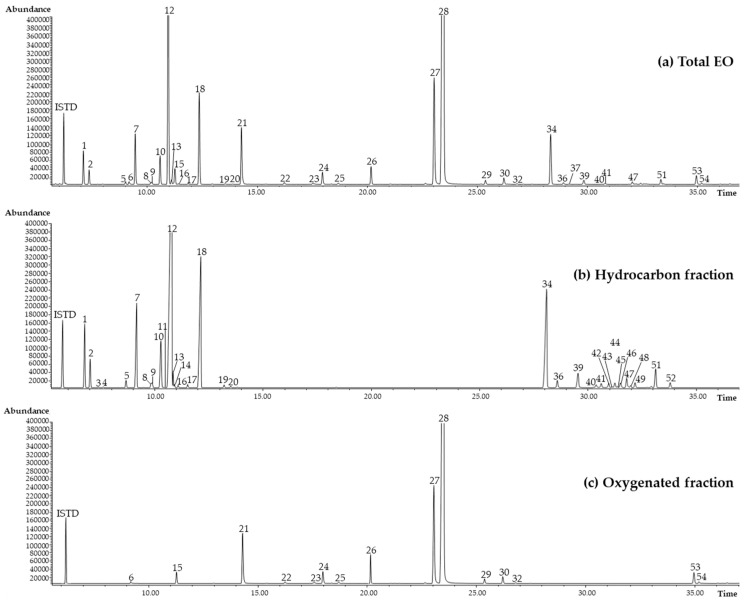
GC-MS profile of (**a**) total *L. origanoides* Kunth EO, (**b**) hydrocarbon and (**c**) oxygenated fractions analyzed in a conventional apolar GC column. Peak numbers refer to Table 1.

In this study, *L. origanoides* Kunth EO, obtained by hydrodistillation from the leaves of botanically authenticated plants from the Embrapa Western Amazon germplasm bank in Manaus (AM), Brazil, was investigated. The essential oil was analyzed using gas chromatography coupled with mass spectrometry (GC-MS) and gas chromatography coupled with a flame ionization detector (GC-FID) to determine its composition, and to identify and quantify all potential constituents of LOEO that contribute to the biological activities studied. The normalized relative percentage abundances (calculated from the absolute areas normalized to the internal standard, nonane, using predicted response factors of all detected compounds) were determined [22,23]. Figure 1a shows the GC-MS profile of LOEO analyzed on a conventional apolar GC column, with peak numbers corresponding to the identified compounds listed in Table 1. As indicated in Table 1, LOEO is characterized by a predominance of oxygenated compounds, with carvacrol as the most abundant component (46.7%), followed by thymol (7.7%). The hydrocarbon fraction is also present in a significant percentage, accounting for more than 35% of the essential oil, with *p*-cymene representing the major hydrocarbon constituent (19.2%).

### 2.2. Enantiomeric Composition of the Investigated EO

The enantiomeric composition of chiral terpenes and terpenoids in LOEO was determined using two chiral columns with cyclodextrin-based stationary phases: 2,3-di-*O*-ethyl-6-*O*-*tert*-butyldimethylsilyl-β-cyclodextrin (DET-Beta) and 2,3-di-*O*-methyl-6-*O*-*tert*-butyldimethylsilyl-β-cyclodextrin (DMT-Beta). This approach minimized enantiomer coelution and overlaps with other compounds, enabling the identification of the enantiomer of several chiral compounds present in a very high enantiomeric abundance, including (1*S*,4*R*)-(−)-camphene, (*S*)-(+)-α-phellandrene, (1*R*,6*S*)-(−)-δ-3-carene, (1*S*,4*S*)-(−)-camphor, (*R*)-(−)-linalool and (1*S*,2*R*,4*S*)-(−)-borneol. In contrast, α-thujene, α-pinene, β-pinene, limonene and terpinen-4-ol were identified as scalemic mixtures, while sabinene was found to be nearly racemic. The complete enantioselective analysis results are presented in Table 2.

### 2.3. In Vitro Inhibitory Activity of the L. origanoides EO Against Acetylcholinesterase, Butyrylcholinesterase, α-Glucosidase, Lipase and Tyrosinase

The inhibitory activity of LOEO was comprehensively evaluated for the first time against five key enzymes, AChE, BChE, α-glucosidase, lipase and tyrosinase, using spectrophotometric assays specifically optimized by the authors.

Table 3 presents the percentage inhibition values of LOEO against the five enzymes, compared with those of well-known reference drugs: galanthamine and rivastigmine for AChE and BChE, acarbose for α-glucosidase, orlistat for lipase and kojic acid for tyrosinase. Notably, LOEO demonstrated inhibitory activity across all enzymes tested, although to variable extents. In particular, the EO displayed a dual inhibitory effect on both AChE and BChE, which are the key therapeutic targets in the treatment of Alzheimer’s disease at both early and advanced stages [26,27]. Its substantial inhibition of α-glucosidase and lipase further highlights its potential as a natural adjuvant in the management of type 2 diabetes and obesity-related metabolic dysfunctions [28,29]. Furthermore, the EO’s inhibition of tyrosinase, a critical enzyme involved in melanin synthesis, suggests promising cosmetic and dermatological applications, particularly in the treatment of hyperpigmentation [30].

To further quantify and compare the potency of LOEO with the respective reference drugs, the inhibitory concentration required to reduce enzyme activity by 50% (IC_50_) was determined for each assay. These results, summarized in Table 4, provide a deeper evaluation of the EO’s bioactivity. LOEO exhibited an IC_50_ against AChE approximately 50 times higher than that of galanthamine, indicating a moderate but relevant and interesting inhibitory effect. Against α-glucosidase, the EO showed an IC_50_ approximately 10 times lower than that of acarbose under the applied assay conditions, retaining a meaningful level of activity that supports its potential role in glucose metabolism regulation. It is worth noting that, while the EO appeared more active than the reference drug, these results reflect the specific experimental conditions used in this study and may not directly be translated to clinical potency; similar findings have also been reported in other studies on other EOs [31,32]. While the inhibition of pancreatic lipase was comparatively less potent, LOEO still exhibited measurable activity, suggesting a possible supportive role in lipid metabolism when used in combination with other agents. For BChE and tyrosinase, although LOEO produced a clear inhibitory effect, 50% inhibition was not reached under the tested conditions, and, thus, IC_50_ values could not be determined.

### 2.4. Contribution of Carvacrol in L. origanoides EO to Experimental Inhibitory Activity: Calculation of Expected Inhibitory Activity

Given the complex mixture of bioactive compounds present in the EO, identifying the specific components responsible for the observed inhibitory effects is fundamental. To attribute the broad enzymatic inhibitory activity of LOEO to its compounds, the role of carvacrol, the major constituent of LOEO, was investigated. The study aimed to determine whether carvacrol alone was responsible for the observed enzymatic inhibition or whether other components contributed to the overall EO biological activity.

**Figure 2:**
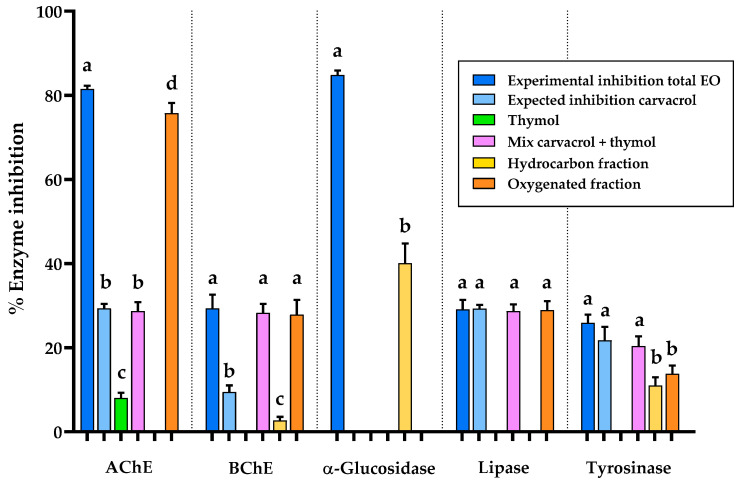
Bar charts comparing the enzymatic inhibition of AChE, BChE, α-glucosidase, lipase, and tyrosinase by *Lippia origanoides* essential oil (EO, blue), the predicted inhibition assuming carvacrol as the sole active compound (light blue), thymol (green), their mixture (violet), and the hydrocarbon and oxygenated fractions obtained by automated flash chromatography (yellow and orange, respectively). Different letters indicate statistically significant differences (*p*-value < 0.05), as determined by Tukey’s post hoc test performed separately for each enzyme.

To assess carvacrol’s contribution, its content in LOEO was quantified as 46.7 g/100 g. The expected inhibitory activity of LOEO was then calculated by assuming that carvacrol was the sole active compound. A dose–response curve for pure carvacrol was generated for each enzyme considered in this study, using the same *in vitro* spectrophotometric assay applied to the EO. The quantified carvacrol concentration in LOEO was converted into the corresponding assay concentration and interpolated on the dose–response curve to estimate the expected inhibitory activity. Curve fitting and interpolation were performed using the same software employed for IC_50_ and EC_50_ determination (see Section 4.9). By comparing the calculated expected inhibition values with the experimentally observed inhibitory activity of the whole LOEO (see Figure 2), it was clear that the inhibitory activity could not be fully attributed to carvacrol for any enzyme, except for lipase, for which carvacrol showed a perfectly aligned experimental total EO inhibition, with the expected inhibition calculated under the assumption that carvacrol was the only active compound, with no statistically significant difference between the two values. While carvacrol contributed partially to the inhibition of most of the other enzymes investigated, it was completely inactive against α-glucosidase. To further investigate the contributions of individual components, the study proceeded with a bio-guided fractionation approach.

### 2.5. Identification of Bioactive Components by Bio-Guided Assay Fractionation

*L. origanoides* EO was further investigated through bio-guided fractionation due to its promising broad-spectrum inhibitory effects. The oxygenated and hydrocarbon EO fractions were isolated using automated flash chromatography and subsequently tested for inhibitory activity against AChE, BChE, α-glucosidase, lipase and tyrosinase. In total, 957.2 ± 7.0 mg of EO was fractionated, yielding 782.5 ± 5.3 mg of the oxygenated fraction and 150.3 ± 8.5 mg of the hydrocarbon fraction (n = 3). GC-MS and GC-FID analyses were performed on each fraction to identify and quantify their chemical compositions. Figure 1b,c show the GC-MS chromatograms of the hydrocarbon and oxygenated fractions from the LOEO fractionation, while Table 1 presents their relative chemical compositions. Both fractions were tested for enzymatic inhibition at concentrations proportional to their abundance within the whole sample. These results make it possible to deduce the specific contributions of the hydrocarbon and oxygenated fractions to the broad LOEO inhibitory profile, beyond carvacrol’s contribution (see Figure 2).

To assess whether the inhibitory activity of the oxygenated fraction could be ascribed solely to carvacrol or whether other structurally related oxygenated constituents were involved, thymol, the second most abundant compound in this fraction and a close structural analogue of carvacrol, was also evaluated. Thymol was tested both individually and in combination with carvacrol at concentrations reflecting their relative proportions in the EO.

For AChE, the observed inhibition could only be partially attributed to carvacrol, and thymol alone exhibited weak inhibitory activity against AChE, while no inhibitory effects were observed for the other enzymes tested. Moreover, the thymol–carvacrol combination produced an inhibitory effect comparable to that of carvacrol alone, with no statistically significant difference between the two, but did not reach the activity of the oxygenated fraction. Notably, the inhibitory profile of the total essential oil was largely reproduced by the oxygenated fraction, not reaching the activity of that of the total EO, indicating that additional oxygenated constituents play an important role.

A similar pattern was observed for BChE, where carvacrol accounted for only a minor portion of the total inhibitory effect of the essential oil. In this case, the thymol–carvacrol mixture displayed an inhibitory activity comparable to that of the oxygenated fraction. In this case, the thymol–carvacrol mixture, the oxygenated fraction, and the total essential oil displayed comparable inhibitory activity, with no statistically significant differences among them. Fractionation nonetheless indicated that the oxygenated fraction largely accounts for the observed activity, while the hydrocarbon fraction showed only a modest contribution, confirming that the principal bioactive compounds are the two major oxygenated constituents, carvacrol and thymol.

In contrast, carvacrol and the oxygenated fraction do not inhibit α-glucosidase, indicating that the activity observed for the EO is attributable to other constituents. Consistently, both thymol alone and the thymol–carvacrol mixture were inactive against α-glucosidase. The hydrocarbon fraction displays an inhibitory effect of 40.1%, although this remains well below the 84.8% inhibition exerted by the total EO. To clarify the origin of this activity, the main hydrocarbon components were evaluated both individually and as mixtures at concentrations reflecting their proportions within the EO. *p*-Cymene and γ-terpinene (accounting for 19.2% and 5.6% of the EO, respectively) were inactive, whereas (*E*)-β-caryophyllene (3.7% of the essential oil) inhibited the enzyme by 5.0%. When the three compounds were combined, the mixture produced 24.6% inhibition, showing the importance of compound combinations within the phytocomplex, as even inactive constituents can contribute to activity when combined, highlighting the complex nature of essential oils. To further assess the possible contribution of minor constituents of the hydrocarbon fraction, α-terpinene and β-myrcene (1.7% and 2.7%, respectively) were also tested. Despite their relatively low concentrations, they inhibited the enzyme by 6.4% and 22.9%, respectively. These five compounds were then combined and tested at concentrations corresponding to their concentration in the EO; the resulting mixture produced 52.0% inhibition. Although this activity did not match that of the total essential oil, it indicates that minor constituents can significantly contribute to the inhibitory effect or modulate the activity of other compounds, as evidenced by the inhibition being higher than that of the total hydrocarbon fraction.

For lipase, the inhibitory effect predicted for carvacrol is consistent with the activity of the total EO, and fractionation confirms that only the oxygenated fraction exerts inhibition. The total EO, the expected inhibition calculated for carvacrol, the thymol–carvacrol mixture, and the oxygenated fraction all showed comparable values, with no statistically significant differences among them. Accordingly, lipase inhibition appears to be largely driven by carvacrol. Tyrosinase inhibition seems to be due exclusively to carvacrol. In fact, thymol alone did not show inhibitory activity, and its combination with carvacrol achieved a result comparable to that of carvacrol alone, indicating no enhancement of activity. Both the hydrocarbon and oxygenated fractions displayed measurable tyrosinase inhibition, each contributing to the overall activity of the essential oil. The inhibitory effect observed for the total EO was consistent with the contributions of both fractions, suggesting that compounds present in both fractions participate in tyrosinase inhibition.

### 2.6. Scavenging Effect on DPPH^•^ and ABTS^+•^ Radicals

After evaluating the broad-spectrum enzymatic inhibitory activity of LOEO, *in vitro* colorimetric assays using DPPH^•^ and ABTS^+•^ were conducted to assess its potential antioxidant activity. The antioxidant properties of phenolic monoterpenoids are well-documented and closely linked to their chemical structures, as phenolic groups can be readily oxidized to quinoid forms, which reduce and scavenge free radicals [33]. As carvacrol, the main compound in LOEO, is a phenolic compound, its scavenging activity was also investigated.

To determine the DPPH^•^ and ABTS^+•^ scavenging activity curves and calculate the EC_50_ values (defined as the concentration required to scavenge 50% of DPPH^•^ or ABTS^+•^ radicals), an online program provided by AAT Bioquest was used.

The EC_50_ values for DPPH^•^ and ABTS^+•^ scavenging for LOEO were 23.1 ± 1.0 μg/mL and 3.0 ± 0.4 μg/mL, respectively. In comparison, Trolox (used as a positive control) showed EC_50_ values of 2.3 ± 0.1 μg/mL and 1.8 ± 0.2 μg/mL, respectively. Therefore, LOEO exhibited an EC_50_ approximately tenfold higher than Trolox in the DPPH assay and about only twofold higher in the ABTS assay.

**Figure 3:**
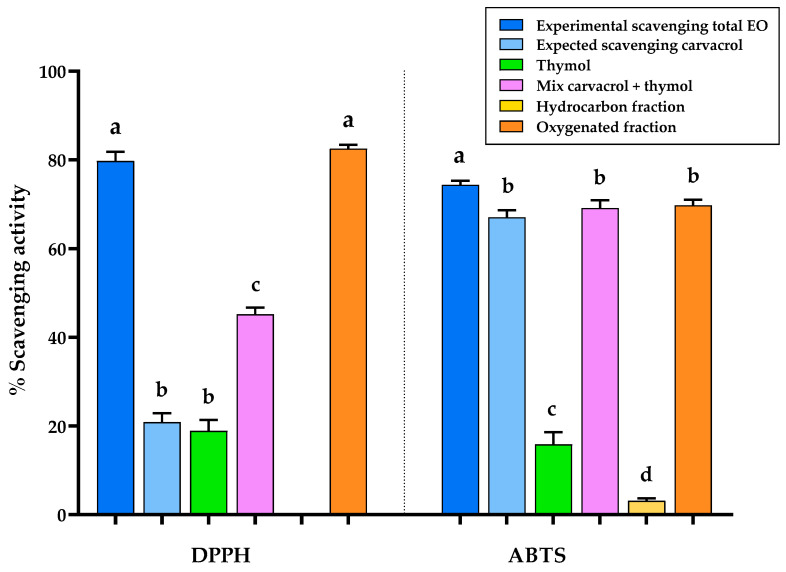
Bar chart comparing DPPH^•^ and ABTS^+•^ scavenging activity of *Lippia origanoides* essential oil (EO, blue; tested at 73.9 μg/mL for DPPH^•^ and 4.8 μg/mL for ABTS^+•^), the predicted activity assuming carvacrol as the sole active compound (light blue), thymol (green), their mixture (violet), and the hydrocarbon and oxygenated fractions obtained by automated flash chromatography (yellow and orange, respectively). Different letters indicate statistically significant differences (*p*-value < 0.05), as determined by Tukey’s post hoc test performed separately for each enzyme.

For the comparative analysis shown in Figure 3, the total essential oil was tested at concentrations higher than the respective EC_50_ values, namely 73.9 μg/mL for the DPPH^•^ assay and 4.8 μg/mL for the ABTS^+•^ assay; the fractions and individual compounds were evaluated at equivalent concentrations relative to the total essential oil, following the same approach adopted for the enzymatic inhibition assays.

As in the enzymatic inhibitory assays, the expected scavenging effect due solely to carvacrol, as the main compound, was calculated as described in Section 2.4. and compared with the experimental results. By analyzing the expected and observed scavenging effects of the total LOEO (see Figure 3), it became evident that the DPPH^•^ scavenging activity could not be entirely attributed to carvacrol. However, for ABTS^+•^, the experimental scavenging effect was close to the expected value calculated under the assumption that carvacrol was the sole active compound, though it was not perfectly aligned and showed a statistically significant difference.

To further investigate the contribution of other constituents to the overall antioxidant activity, the fractions obtained by flash chromatography, as well as thymol and the carvacrol–thymol mixture, were evaluated following the same approach used for the enzymatic assays. As shown in Figure 3, in the DPPH^•^ assay, the oxygenated fraction was the only active fraction, displaying scavenging activity comparable to that of the total essential oil, with no statistically significant difference between them. This finding suggests that additional oxygenated compounds, likely sharing structural features with carvacrol, contribute to the scavenging effect. This interpretation is supported by the additional tests, as thymol alone, despite being present in the EO at a lower concentration than carvacrol, exhibited comparable scavenging activity. Moreover, the activity of the carvacrol–thymol mixture appeared additive but did not reach the scavenging capacity of the oxygenated fraction, indicating that other oxygenated constituents also play a role. In this context, compounds such as α-terpineol and 1,8-cineole, previously reported in the literature to possess antioxidant activity [34], may contribute to the overall effect. In the ABTS^+•^ assay, the oxygenated fraction was the principal contributor to scavenging activity. Also in this case, thymol was confirmed to participate in the antioxidant activity, and in combination with carvacrol, its scavenging effect was comparable to that of the oxygenated fraction, with no statistically significant difference between them.

## 3. Discussion

The results of this study provide a comprehensive picture of the chemical complexity and multifunctional biological potential of *L. origanoides* EO, highlighting the importance of the phytocomplex in driving bioactivity. The predominance of oxygenated monoterpenes, particularly carvacrol and thymol, in the composition of LOEO is consistent with data reported in the literature [2,35] and indicates that the plant material used for isolation belongs to the carvacrol chemotype. Plants of this species are generally classified into five chemotypes according to the dominant constituents of their essential oil: chemotype A, characterized by *p*-cymene, (*E*)-β-caryophyllene, α- and β-phellandrene, limonene, α-humulene and 1,8-cineole, and associated with a citrus-like odor; chemotype B, dominated by carvacrol; chemotype C, dominated by thymol, with both B and C exhibiting the typical “oregano” scent; chemotype D, defined by high 1,8-cineole levels and a fresh, camphoraceous odor; and chemotype E, characterized by (*E*)-methyl cinnamate and (*E*)-nerolidol, which impart a fruity–woody fragrance reminiscent of cinnamon, strawberry and wood [36,37].

The results obtained clearly demonstrate that carvacrol alone cannot account for most of the biological effects, with the notable exception of lipase inhibition, indicating that synergistic or additive interactions among multiple constituents are crucial. This hypothesis is further reinforced by bio-guided fractionation, which revealed a clear functional differentiation between the oxygenated and hydrocarbon fractions. The oxygenated fraction largely drove AChE, BChE, lipase inhibition, DPPH^•^ and ABTS^+•^ scavenging, whereas α-glucosidase inhibition was primarily associated with the hydrocarbon fraction, despite the inactivity of major hydrocarbons when tested individually. The enhanced activity observed when minor hydrocarbons were combined underscores the role of low-abundance constituents in modulating enzyme inhibition, supporting the presence of synergistic or additive interactions that emerge only within the phytocomplex. Tyrosinase inhibition involved contributions from both fractions, reflecting a more complex interaction pattern. Notably, except for AChE and tyrosinase inhibition, all the enzymatic activities investigated in this study (including BChE, α-glucosidase, and lipase inhibition) are reported here for the first time for LOEO, thus expanding current knowledge on its biological potential. To date, no studies have described BChE, lipase, or α-glucosidase inhibitory activities for *L. origanoides* essential oil, although such effects have been reported for other plant extracts of the same species. Regarding tyrosinase inhibition, a previous study evaluated different chemotypes of *L. origanoides*, but did not include the carvacrol chemotype investigated here, thus preventing a direct comparison [38]. In this context, the AChE inhibitory activity observed in this study (IC_50_ = 22.9 μg/mL) closely matches previously reported data for LOEO (IC_50_ ≈ 19.3 μg/mL), confirming the consistency and reliability of the anticholinesterase potential of this species despite differences in experimental conditions [20,21]. Furthermore, the literature indicates that phenolic monoterpenoids such as carvacrol and thymol play a key role in AChE inhibition, consistent with the activity trends observed in this work [20,21].

Within this framework, thymol was selected as a representative compound due to its structural similarity to carvacrol, allowing a targeted evaluation within the same chemical class. This choice was intended to assess whether structurally related oxygenated monoterpenes contribute comparably to the observed activities, rather than systematically screening all individual constituents. The evaluation of thymol, both individually and in combination with carvacrol, further supports the hypothesis that the biological activity of the oxygenated fraction cannot be explained solely by the presence of a single dominant compound. While thymol alone exhibited only limited activity against AChE, its combination with carvacrol reproduced the inhibition patterns observed for the oxygenated fraction in several assays, indicating that structurally related monoterpenoids may act cooperatively within the phytocomplex.

Importantly, the unprecedented enantiomeric profiling adds a new dimension to the understanding of LOEO bioactivity, as stereochemistry may influence enzyme interactions and antioxidant behavior. In fact, enantiomers, as widely recognized in the literature, are molecular entities with identical chemical structures that exist as non-superimposable mirror images of each other. Despite their structural similarity, enantiomers often display distinct biological activities [39]. In biological systems, whether plant or animal, processes are frequently stereoselective, resulting in the formation of chiral components with a high enantiomeric excess [40], but can also produce scalemic or racemic mixtures. Therefore, investigating the enantiomeric composition and recognition of these chiral components is essential for gaining deeper insights into the matrix and its biological behaviors.

Collectively, these results demonstrate that although carvacrol plays an important role in several enzymatic activities, the biological profile of LOEO cannot be attributed to a single dominant constituent. Instead, its broad inhibitory spectrum arises from the combined contributions of both hydrocarbon and oxygenated compounds. These findings highlight the importance of considering the phytocomplex when evaluating the EO biological properties, rather than focusing solely on their major components.

Future research on *L. origanoides* EO should focus on clarifying the roles of minor constituents and their synergistic interactions, particularly those responsible for AChE, α-glucosidase and tyrosinase inhibition. In addition, *in vivo* and cellular models, together with advanced formulation strategies, will be fundamental to assess bioavailability, safety and applicability in therapeutic, nutraceutical, and cosmetic contexts.

## 4. Materials and Methods

### 4.1. Essential Oil Isolation

*L. origanoides* Kunth samples were obtained from Embrapa Western Amazon germplasm bank in Manaus (AM), Brazil. The plant leaves were subjected to hydrodistillation in a Clevenger-type apparatus for 2 h. Access to the Brazilian biodiversity was authorized by CGEN under registry AC6AC63. The EO was collected and stored in the dark at +4 °C until use.

### 4.2. Reagents

Acetylcholinesterase (AChE) from *Electrophorus electricus* L., butyrylcholinesterase (BChE) from equine serum, α-glucosidase from *Saccharomyces cerevisiae* Meyen ex E.C. Hansen, lipase from porcine pancreas, mushroom tyrosinase, acetylthiocholine iodide (ATCI), butyrylthiocholine iodide (BTCI), 5,5-dithiobis-2-nitrobenzoic acid (DTNB), *p*-nitrophenyl-α-D-glucopyranoside (*p*-NPG), *p*-nitrophenyl palmitate, L-tyrosine, acarbose, galanthamine hydrobromide, rivastigmine tartrate, orlistat, dimethyl sulfoxide (DMSO), acetonitrile (≥99.0%), ethanol (≥99.8%), cyclohexane (≥99.5%), nonane (≥99.0%), petroleum ether (min 75%), ethyl acetate (≥99.5%), carvacrol (≥98.0%) and all the buffer components (TWEEN20, bovine serum albumin, Trizma base, Trizma HCl, MgCl_2_•6H_2_O, NaCl, NaH_2_PO4, Na_2_HPO_4_, Na_2_CO_3_) were purchased from Merck Life Science S.r.l. (Milan, Italy).

### 4.3. In Vitro Enzymes Inhibition Tests

All assays were performed in a sealed 4 mL vial (1.5 mL for the tyrosinase assay) to prevent the loss of any EO components into the surrounding environment and to minimize their release into the head space above the reaction mixture. The assays were optimized for essential oil matrices, particularly by adjusting solvent conditions (DMSO content), substrate/enzyme ratios, and assay timing to ensure measurements within the linear kinetic range. The percentage inhibition of AChE, BChE, α-glucosidase, lipase and tyrosinase was measured according to the equation below:

$$\%\;Inhibition=\frac{∆A\left(Control\right)-∆A(Sample)}{∆A\left(Control\right)}\times 100$$

where:

∆*A (Control)* = *A Control* − *A Control Blank*

∆*A(Sample)* = *A Sample* − *A Sample Blank*

#### 4.3.1. *In Vitro* AChE and BChE Inhibition Test

The protocol used in this study was optimized by Pavarino et al. [41] by modifying that of Rhee et al. [42]. The buffers used in the experiment were: buffer A (50 mM Tris-HCl, pH 8), buffer B (50 mM Tris-HCl, pH 8, supplemented with 0.1% bovine albumin and 0.1% TWEEN20) and buffer C (50 mM Tris-HCl, pH 8, containing 0.1 M NaCl and 0.02 M MgCl_2_•6H_2_O). The solutions for both enzymes (0.22 U/mL) were prepared using buffer B. Acetylthiocholine iodide (ATCI) at 1.5 mM was dissolved in Millipore water, while butyrylthiocholine iodide (BTCI) was dissolved in buffer A. Ellman’s reagent solution (3 mM) was prepared in buffer C. Stock solutions of essential oil (15 mg/mL) and galanthamine (0.4 mg/mL) and rivastigmine (50 mg/mL), used as positive controls, were dissolved in dimethyl sulfoxide (DMSO).

The reaction mixture was prepared in the following order: 150 µL of ATCI or BTCI solution; 750 µL of Ellman’s reagent solution; 900 µL of buffer B; 5 µL of essential oil stock solution, single compound, EO fraction or galanthamine hydrobromide/rivastigmine tartrate solution; and 150 µL of the enzyme solution. The final concentration of DMSO in the reaction mixture was 0.3%. The mixture was incubated at 30 °C for 6 min, after which absorbance was measured at 405 nm.

For the control solution, in which enzymes remained fully active, 5 µL of pure DMSO replaced the sample solution. Control and sample blanks were prepared by substituting 150 µL of enzyme solution with 150 µL of buffer A.

#### 4.3.2. *In Vitro* α-Glucosidase Inhibition Test

The protocol used in this study was optimized by the authors by modifying that of Oboh et al. [43]. The buffer used in the experiment was 0.1 M sodium phosphate buffer (pH 6.9). The α-glucosidase solution (0.125 U/mL) and *p*-NPG solution (2.5 mM) were prepared in sodium phosphate buffer and freshly renewed daily. EO (0.5 mg/mL) and acarbose (50 mg/mL) stock solutions, used as positive controls, were prepared in DMSO.

The reaction mixture was prepared in the following order: 1 mL of sodium phosphate buffer; 100 µL of EO stock solution, single compound, EO fraction or acarbose solution; 320 µL of α-glucosidase solution; and finally 160 µL of *p*-NPG solution. The final concentration of DMSO in the reaction mixture was 6.3%. The mixture was incubated at 37 °C for 10 min. After incubation, 400 µL of 0.2 M Na_2_CO_3_ in Millipore water was added to stop the reaction. The absorbance was then measured at 405 nm, which enabled the identification of *p*-nitrophenol.

For the control solution, in which α-glucosidase remained fully active, 100 µL of pure DMSO replaced the sample solution. Control and sample blanks were prepared by substituting 320 µL of the enzyme solution with 320 µL of sodium phosphate buffer.

#### 4.3.3. *In Vitro* Lipase Inhibition Test

The protocol used in this study was optimized by the authors by modifying that of Slanc et al. [44]. The buffer used in the experiment was 75 mM Tris-HCl (pH 8.5). The lipase solution (0.6 mg/mL) from porcine pancreas was prepared in Tris-HCl buffer, while the *p*-nitrophenyl palmitate solution (6.6 mM) was prepared in an acetonitrile/ethanol mixture (1:3 *v*/*v*). Both solutions were freshly renewed daily. EO (5 mg/mL) and orlistat (0.01 mg/mL) stock solutions, used as a positive controls, were prepared in DMSO.

The reaction mixture was assembled in the following order: 930 µL of Tris-HCl buffer; 50 µL of *p*-nitrophenyl palmitate solution; 20 µL of EO stock solution, single compound, EO fraction or orlistat solution; and finally, 1 mL of lipase solution. The final concentration of DMSO in the reaction mixture was 1%. The mixture was incubated at 37 °C for 30 min.

After incubation, the absorbance was measured at 405 nm, a wavelength used for the identification of *p*-nitrophenol. For the control solution, in which lipase remained fully active, 20 µL of pure DMSO replaced the sample solution. Control and sample blanks were prepared by substituting 1 mL of the enzyme solution with 1 mL of Tris-HCl buffer.

#### 4.3.4. *In Vitro* Tyrosinase Inhibition Test

The protocol used in this study was the one optimized in a previous work by some of the authors [45]. The mushroom tyrosinase solution (200 U/mL) from *Agaricus bisporus* (J.E. Lange) Imbach was prepared in sodium phosphate buffer (pH 6.8), stored in 9 mL aliquots at −18 °C and thawed just before use. The L-tyrosine solution (0.1 mg/mL) was freshly prepared daily in sodium phosphate buffer (pH 6.8). EO (2.5 mg/mL) and kojic acid stock solution (0.02 mg/mL), used as a positive control, were prepared in DMSO. The reaction mixture was assembled in the following order: 400 µL of mushroom tyrosinase solution; 500 µL of sodium phosphate buffer; 100 µL of EO stock solution, single compound, EO fraction or kojic acid solution; and finally, 500 µL of L-tyrosine solution. The final concentration of DMSO in the reaction mixture was 6.6%. The mixture was incubated at 25 °C for 6 min.

After incubation, absorbance was measured at 475 nm, a wavelength used for the identification of dopachrome. For the control solution, in which tyrosinase remained fully active, 100 µL of pure DMSO replaced the sample solution. Control and sample blanks were prepared by substituting 400 µL of the enzyme solution with 400 µL of sodium phosphate buffer.

### 4.4. Scavenging Effect on DPPH^•^ Radicals

The capacity to scavenge the free radical DPPH^•^ was monitored using the method reported by Mastellone et al. [46] with some modifications. A solution of the DPPH^•^ radical (25 μg/mL) was prepared in methanol. The EO and its fractions were dissolved in DMSO, and 30 μL of this solution (5 mg/mL) was combined with 2 mL of the reagent. The mixture was thoroughly shaken and kept in the dark at room temperature for 75 min until the absorbance stabilized. The reduction of the DPPH^•^ radical was assessed by measuring the decrease in absorbance at 515 nm.

The DPPH^•^ scavenging effect at each concentration was determined using the following equation:

$$\%\;Scavenging\;effect=\frac{\left[\left(A_{DPPH}-A_{S}\right)\right]}{A_{DPPH}}\times 100$$

where *A_DPPH_* is the absorbance of the DPPH^•^ solution and *A_S_* is the absorbance after the sample was added. Various concentrations of LOEO were prepared to generate a dose–response curve, and the essential oil concentration required to achieve a 50% scavenging effect (EC_50_) was determined. The same method was used to determine the EC_50_ of Trolox, a well-known phenolic antioxidant, which served as a positive control to validate the experiment.

### 4.5. Scavenging Effect on ABTS^+•^ Radicals

The capacity to scavenge the free radical ABTS^+•^ was monitored using the method reported by Mastellone et al. [46] with some modifications. To generate the ABTS^+•^ radical, 5 mL of K_2_S_2_O_8_ solution (0.66 mg/mL) was mixed with 5 mL of ABTS (3.84 mg/mL), and the resulting solution was kept in the dark at −20 °C for 16 h. This solution was then diluted with an ethanol/water mixture (50:50 *v*/*v*) to prepare the working reagent. Once the radical was formed, 2 mL of the ABTS^+•^ solution was added to 100 μL of the EO (0.1 mg/mL) or its fraction solution in DMSO. The absorbance at 734 nm was recorded after 6 min to evaluate the scavenging activity.

The ABTS^+•^ scavenging effect for each sample was calculated using the following equation:

$$\%\;Scavenging\;effect=\frac{\left[\left(A_{ABTS}-A_{S}\right)\right]}{A_{ABTS}}\times 100$$

where *A_ABTS_* is the absorbance of the ABTS^+•^ solution and *A_S_* is the absorbance after sample was added. Various concentrations of LOEO were prepared to generate a dose–response curve, and the essential oil concentration required to achieve a 50% scavenging effect (EC_50_) was determined. The same method was used to determine the EC_50_ of Trolox, a well-known phenolic antioxidant, which served as a positive control to validate the experiment.

### 4.6. Flash Column Chromatography

Fractionation was carried out using a PuriFlash 450 flash column chromatography system (Sepachrom, Milan, Italy), equipped with both UV and Evaporative Light Scattering (ELSD) detectors. The mobile phase consisted of petroleum ether (solvent A) and ethyl acetate (solvent B), with a linear elution gradient from 100% A to 70% A and 30% B over 20 min. The hydrocarbon fraction was eluted during the initial isocratic phase with 100% petroleum ether, while the oxygenated fraction was eluted during the gradient phase as the concentration of ethyl acetate increased. Pre-packed Sphera silica cartridges (50 µm, Sepachrom, Italy) were used, with a constant eluent flow rate of 25 mL/min. Fractionation was performed on three replicates.

To evaluate the enzymatic inhibitory activity of the hydrocarbon and oxygenated fractions, each fraction was tested at a concentration reflecting its relative abundance in the EO, as determined by GC-FID analysis. This approach ensured that the fractions were assessed in the same proportions as they naturally occur in the total EO.

### 4.7. GC-FID and GC-MS Analysis Conditions

The EO solution and its respective fractions were prepared in cyclohexane at a concentration of 5.0 mg/mL. A solution of nonane in cyclohexane (20 mg/mL) was added as an internal standard (5 μL) and the samples were analyzed by GC-MS and GC-FID.

GC-MS analyses were performed using an Agilent 6890 GC system coupled to an Agilent 5975 MSD (Agilent, Little Falls, DE, USA), equipped with an MPS-2 multipurpose sampler (Gerstel, Mülheim a/d Ruhr, Germany). Data processing was carried out using Agilent ChemStation software (release F.01.03.2357).

GC-FID analyses were conducted on a Shimadzu GC-2010 system (Shimadzu, Milan, Italy) equipped with GC Solution software (version 2.53SU).

The chromatographic conditions for compound identification (MS) and quantification (FID) were as follows: injector temperature: 250 °C; injection mode: split; split ratio: 1/20; injection volume: 1 μL; carrier gas: helium; constant flow rate: 1 mL/min; column: MEGA-5 MS (95% polydimethylsiloxane, 5% phenyl) 30 m, 0.25 µm *d_f_* × 0.25 mm *d_c_* from MEGA S.r.l. (Legnano, Milan, Italy). Elution was conducted according to the following temperature program: 50 °C (1 min)—3 °C/min—250 °C (5 min).

EO compounds were identified by comparing their linear retention indices (*I^T^*s), calculated with a C_9_-C_25_ hydrocarbon mixture, and their mass spectra with those of authentic standards and/or from commercially available mass spectral libraries [24]. MSD conditions: MS operated in EI mode (70 eV); scan range: 35–350 amu; dwell time: 34 ms; ion source temperature: 250 °C; quadrupole temperature: 150 °C; transfer line temperature: 270 °C.

EO components were quantified using GC-FID by internal standardization. Nonane was used as the internal standard, and the peak areas from the FID detector were normalized to the area of the internal standard. Corrections were applied using predicted response factors [22,23], following the methodology of Cachet et al. [23]. Results are expressed as g/100 g of EO.

### 4.8. Enantioselective Analyses (GC-MS)

The EO solutions were prepared under the same conditions as those used for quali-quantitative GC-MS and GC-FID analysis (see Section 4.7). GC-MS analyses were performed using a Shimadzu GC-MS system, consisting of a Shimadzu GC2010 gas chromatograph connected to a Shimadzu QP2010 Plus mass spectrometer (Shimadzu, Milan, Italy). An MPS2 MultiPurpose Sampler (Gerstel, Mülheim a/d Ruhr, Germany) served as the autosampler. Capillary columns (25 m, 0.25 µm *d_f_* × 0.25 mm *dc*) coated with cyclodextrin derivatives as stationary phases were used to separate chiral compounds. The two stationary phases used were as follows: 30% 2,3-di-*O*-ethyl-6-*O*-*tert*-butyldimethylsilyl-β-cyclodextrin in PS089 (DET-Beta) and 30% 2,3-di-*O*-methyl-6-*O*-*tert*-butyldimethylsilyl-β-cyclodextrin in PS089 (DMT-Beta). Both columns were from MEGA S.r.l. (Legnano, Italy). The analysis conditions for these columns were as follows: injector temperature: 220 °C, injection mode: split; split ratio: 20:1; injection volume: 1 μL; carrier gas: helium; constant flow rate: 1.0 mL/min (pressure 39.9 kPa). Elution was performed according to the following temperature program: 50—2 °C/min—220 °C (10 min). The enantiomers were identified by matching their spectral similarity with data from both commercial and in-house databases, and their stereochemistry was verified by comparing their linear retention indices (*I^T^*s) to those of authentic standards in a dedicated in-house retention index database, applying a tolerance of ±3 [39].

### 4.9. IC_50_ and EC_50_ Calculation

For the IC_50_ and EC_50_ calculations, an online program from AAT Bioquest was used https://www.aatbio.com/tools/ic50-calculator, accessed on 3 March 2026). The same tool was used to generate fitted dose–response curves and to interpolate the expected inhibitory activity values of carvacrol based on its concentration in LOEO.

### 4.10. Statistical Analysis

Analysis of variance (ANOVA) was performed using GraphPad Prism v8.02 (GraphPad Software, San Diego, CA, USA). One-way ANOVA and post hoc comparison of multiple means (Tukey’s range test) were performed, and statistical significance was assumed at *p*-value < 0.05.

## Figures and Tables

**Table 1:** Chemical composition of *L. origanoides* Kunth essential oil, expressed in g/100 g, including the compositions of the hydrocarbon (HYDR) and oxygenated (OXY) fractions. Analytical relative standard deviation (RSD%) < 3%. Legend: tr: trace.

Peak	Compounds	Exp*I*^T^_s_	Lit*I*^T^_s_ ^‡^ [24]	EO (g/100 g)	HYDR	OXY
1	α-thujene	925	924	1.6	4.1	-
2	α-pinene	932	932	0.7	1.9	-
3	camphene	949	946	-	0.1	-
4	sabinene	973	972	-	tr	-
5	β-pinene	978	974	0.2	0.5	-
6	1-octen-3-ol	980	974	-	-	0.2
7	β-myrcene	990	988	2.7	6.7	-
8	α-phellandrene	1008	1002	0.1	0.3	-
9	δ-3-carene	1009	1008	0.1	0.4	-
10	α-terpinene	1018	1014	1.7	4.3	-
11	*m*-cymene *	1022	1021	-	0.1	-
12	*p*-cymene *	1025	1024	19.2	45.2	-
13	limonene	1030	1024	0.3	0.6	-
14	β-phellandrene	1033	1025	-	0.2	-
15	1,8-cineole	1034	1026	1.0	-	1.0
16	(*Z*)-β-ocimene	1037	1032	tr	0.1	-
17	(*E*)-β-ocimene	1048	1044	tr	0.2	-
18	γ-terpinene	1059	1054	5.6	14.0	-
19	α-terpinolene	1087	1086	tr	0.2	-
20	*p*-cymenene	1093	1089	tr	0.1	-
21	linalool	1104	1098	3.8	-	4.8
22	ipsdienol	1142	1140	0.1	-	0.2
23	umbellulone	1169	1167	0.1	-	0.2
24	terpinen-4-ol	1178	1174	0.8	-	1.1
25	α-terpineol	1189	1186	0.1	-	0.2
26	thymol methyl ether	1233	1232	1.1	-	3.1
27	thymol	1293	1289	7.7	-	19.1
28	carvacrol	1303	1298	46.7	-	67.3
29	thymol acetate	1349	1355	0.3	-	0.4
30	carvacrol acetate	1369	1371	0.4	-	0.7
31	α-copaene	1374	1374	-	0.1	-
32	geranyl acetate	1382	1381	tr	-	0.1
33	β-elemene	1389	1389	-	0.1	-
34	(*E*)-β-caryophyllene	1418	1417	3.7	12.0	-
35	β-gurjunene	1429	1431	tr	0.1	-
36	(*E*)-α-bergamotene	1437	1432	0.1	0.7	-
37	α-guaiene	1439	1437	tr	0.2	-
38	guaia-6,9-diene	1441	1442	-	0.1	-
39	α-humulene	1455	1452	0.3	1.5	-
40	α-amorphene	1475	1475	tr	0.4	-
41	γ-muurolene	1479	1478	tr	0.5	-
42	β-selinene	1488	1489	tr	0.4	-
43	γ-amorphene	1492	1495	-	0.1	-
44	α-selinene	1495	1498	-	0.4	-
45	α-muurolene	1498	1500	-	0.1	-
46	(*Z*)-α-bisabolene	1501	1503	tr	0.4	-
47	β-bisabolene	1508	1505	0.1	0.9	-
48	(*E*)-γ-cadinene	1513	1513	-	0.2	-
49	δ-cadinene	1518	1522	tr	0.4	-
50	(*E*)-α-bisabolene	1537	1538	0.3	1.7	-
51	germacrene B	1558	1559	-	0.5	-
52	caryophyllene oxide	1582	1582	0.6	-	1.2
53	2-phenylethyl tiglate	1588	1584	tr	-	0.1
54	7-epi-α-eudesmol	1657	1662	tr	-	0.2
	Monoterpenes			32.2		
	Oxygenated monoterpenoids			62.1		
	Sesquiterpenes			4.5		
	Oxygenated sesquiterpenoids			0.6		
	Total			99.4	99.9	99.9

‡ From Adams’ book, ref. [24]. * From Bizzo et al. [25].

**Table 2:** Percentage enantiomeric composition (ec%) of the chiral compounds detected in *L. origanoides* Kunth EO obtained on the two chiral columns with cyclodextrin-based stationary phases, DET-Beta and DMT-Beta. NR: Not Resolved; *: literature retention index not available.

Compounds	Reference Ion (*m*/*z*)	Exp*I*^T^_s_	Lit*I*^T^_s_	DET-Beta (ec%)	Exp*I*^T^_s_	Lit*I*^T^_s_	DMT-Beta (ec%)
(*S*)-α-thujene	93	913	914	66.0	955	*	64.8
(*R*)-α-thujene		915	917	34.0	959	*	35.2
(1*S*,5*S*)-(−)-α-pinene	93	924	923	39.5	967	968	39.4
(1*R*,5*R*)-(+)-α-pinene		922	921	60.5	982	983	60.6
(1*R*,5*R*)-(+)-β-pinene	93	945	944	39.0	1009	1011	39.9
(1*S,*5*S*)-(−)-β-pinene		955	955	61.0	1011	1014	60.1
(1*R*,5*R*)-(+)-sabinene	93	973	972	44.5	1027	1028	44.5
(1*S*,5*S*)-(−)-sabinene		987	988	55.5	1033	1037	55.5
(1*S*,4*R*)-(−)-camphene	93	918	917	92.2	977	977	92.6
(1*R*,4*S*)-(+)-camphene		933	932	7.8	998	999	7.4
(*R*)-(−)-α-phellandrene	93	1017	1017	6.1	1044	1044	6.0
(*S*)-(+)-α-phellandrene		1021	1020	93.9	1047	1048	94.0
(1*R*,6*S*)-(−)-δ-3-carene	93	1027	1027	>99	1061	1054	>99
(*S*)-(−)-limonene	68	1056	1056	44.1	1081	1082	45.6
(*R*)-(+)-limonene		1072	1072	55.9	1096	1097	54.3
(1*S*,4*S*)-(−)-camphor	95	1135	1133	>95	1193	1192	>95
(*R*)-(−)-linalool	71	1175	1174	94.4	1201	1201	94.7
(*S*)-(+)-linalool		1190	1189	5.6	1212	1211	5.3
(1*S*,2*R*,4*S*)-(−)-borneol	95	1195	1192	98.0	1287	1287	97.9
(1*R*,2*S*,4*R*)-(+)-borneol		1202	1200	2.0	1295	1294	2.1
(*S*)-(+)-terpinen-4-ol	71	1252	1248	68.1	1298	1300	NR
(*R*)-(−)-terpinen-4-ol		1256	1253	31.9			

**Table 3:** Percent inhibition of AChE, BChE, α-glucosidase, lipase and tyrosinase of *L. origanoides* EO samples compared to their respective reference drugs used as positive controls for each enzyme, together with their standard deviation (σ).

Samples	Concentration Tested in Reaction Mixture	% Enzyme Inhibition (n = 3)	σ
Galanthamine tested on AChE	1.0 µg/mL	68.2	2.9
Rivastigmine tested on AChE	255.8 µg/mL	58.1	1.5
*L. origanoides* EO tested on AChE	38.4 µg/mL	81.5	0.8
Galanthamine tested on BChE	1.0 µg/mL	24.7	0.6
Rivastigmine tested on AChE	6.4. µg/mL	61.5	3.8
*L. origanoides* EO tested on BChE	38.4 µg/mL	29.3	0.4
Acarbose	126.0 µg/mL	42.0	2.3
*L. origanoides* EO tested on α-Glucosidase	31.6 µg/mL	84.8	1.1
Orlistat	10.0 ng/mL	60.5	3.1
*L. origanoides* EO tested on Lipase	50.0 µg/mL	29.1	2.3
Kojic acid	1.7 µg/mL	23.6	1.1
*L. origanoides* EO tested on Tyrosinase	166.7 µg/mL	25.9	2.0

**Table 4:** IC_50_ values of *L. origanoides* EO and of the relative positive controls for each enzyme together with their standard deviation (σ).

Enzyme	Samples	IC50 (μg/mL)	σ
AChE	Galanthamine	0.468	0.002
	Rivastigmine	206.6	13.7
	*L. origanoides* EO	22.9	0.9
BChE	Galanthamine	3.40	0.02
	Rivastigmine	3.9	0.2
	*L. origanoides* EO	Activity lower than 50%	
α-Glucosidase	Acarbose	154.2	8.4
	*L. origanoides* EO	14.6	0.6
Lipase	Orlistat	0.008	0.001
	*L. origanoides* EO	74.9	3.8
Tyrosinase	Kojic acid	0.509	0.001
	*L. origanoides* EO	Activity lower than 50%	

## Data Availability

The raw data supporting the conclusions of this article will be made available by the authors on request.

## Abbreviations

The following abbreviations are used in this manuscript:

EO	Essential Oil
LOEO	*Lippia origanoides* Kunth Essential Oil
AD	Alzheimer’s disease
AChE	Acetylcholinesterase
BChE	Butyrylcholinesterase
SCCS	Scientific Committee on Consumer Safety
GC	Gas chromatography
MS	Mass Spectrometry
FID	Flame Ionization Detector
DET-Beta	2,3-di-*O*-ethyl-6-*O*-*tert*-butyldimethylsilyl-β-cyclodextrin
DMT-Beta	2,3-di-*O*-methyl-6-*O*-*tert*-butyldimethylsilyl-β-cyclodextrin
DPPH	2,2-difenil-1-picrilidrazile
ABTS	Acido 2,2′-azino-bis(3-etilbenzotiazolin-6-solfonico)
CGEN	Conselho de Gestão do Patrimônio Genético
ATCI	Acetylthiocholine iodide
BTCI	Butyrylthiocholine iodide
DTNB	5,5-dithiobis-2-nitrobenzoic acid
*p*-NPG	*p*-nitrophenyl-α-D-glucopyranoside
DMSO	Dimethyl sulfoxide
EI	Electron Ionization
MSD	Mass Selective Detector
